# *CCR4*, *CCR8*, and *P2RY14* as Prognostic Factors in Head and Neck Squamous Cell Carcinoma Are Involved in the Remodeling of the Tumor Microenvironment

**DOI:** 10.3389/fonc.2021.618187

**Published:** 2021-02-22

**Authors:** Liangliang Meng, Xiaoxi He, Quan Hong, Bo Qiao, Xiao Zhang, Bin Wu, Xiaobo Zhang, Yingtian Wei, Jing Li, Zhaoxiang Ye, Yueyong Xiao

**Affiliations:** ^1^Medical School of Chinese PLA, Beijing, China; ^2^Department of Radiology, The First Medical Centre, Chinese PLA General Hospital, Beijing, China; ^3^Department of Radiology, Chinese PAP Beijing Corps Hospital, Beijing, China; ^4^Department of Radiology, Tianjin Medical University Cancer Institute and Hospital, National Clinical Research Center for Cancer, Tianjin’s Clinical Research Center for Cancer, Key Laboratory of Cancer Prevention and Therapy, Tianjin, China; ^5^Department of Nephrology, The First Medical Center, Chinese PLA General Hospital, Chinese PLA Institute of Nephrology, State Key Laboratory of Kidney Diseases, National Clinical Research Center of Kidney Diseases, Beijing, China; ^6^Department of Stomatology, The First Medical Center, Chinese PLA General Hospital, Beijing, China

**Keywords:** tumor microenvironment, squamous cell carcinoma, survival, immune, gene

## Abstract

The tumor microenvironment (TME) plays a critical role in the initiation and progression of cancer. However, the specific mechanism of its regulation in head and neck squamous cell carcinoma (HNSCC) remains unclear. In this study, we first applied the ESTIMATE method to calculate the immune and stromal scores in patients’ tumor tissues from The Cancer Genome Atlas (TCGA) database. GSE41613, GSE30784, and GSE37991 data sets from the Gene Expression Omnibus (GEO) database were recruited for further validation. Differentially expressed genes (DEGs) were identified and then analyzed by Cox regression analysis and protein-protein interaction (PPI) network construction. DEGs significantly associated with prognosis and TME will be identified as hub genes. These genes were also validated at the protein level by immunohistochemical analysis of 10 pairs of primary tumor tissues and the adjacent normal tissues from our institution. The relationship between hub genes expression and immune cell fraction estimated by CIBERSORT software was also examined. 275 DEGs were significantly associated with TME. *CCR4, CCR8*, and *P2RY14* have then identified as hub genes by intersection Cox and PPI analysis. Further investigation revealed that the expression of *CCR4, CCR8*, and *P2RY14* was negatively correlated with clinicopathological characteristics (clinical stage, T stage) and positively associated with survival in HNSCC patients, especially in male patients. The expression of *CCR8* and *P2RY14* was lower in males than in females. *CCR8* and *P2RY14* were differentially expressed in tumor tissues than normal tissues, and the results were validated at the protein level by immunohistochemistry experiments. Gene set enrichment analysis (GSEA) showed that the high expression groups’ hub genes were mainly enriched for immune-related activities. In the low-expression groups, genes were primarily enriched in metabolic pathways. CIBERSORT results showed that the expression of these genes was all negatively correlated with the fraction of memory B cells and positively correlated with the fraction of the other four cells, including naive B cells, resting T cells CD4 memory, T cells follicular helper, and T cells regulatory (Tregs). The results suggest that *CCR4, CCR8*, and *P2RY14* may be responsible for maintaining the immune dominance of TME, thus leading to a better prognosis.

## Introduction

The most common pathologic type of tumor occurring in the head and neck region is mucosal squamous cell carcinoma, which mainly affects the oral mucosa, palate, tongue, oropharynx, hypopharynx, larynx, and other parts (1). The global incidence of head and neck squamous cell carcinoma (HNSCC) has risen significantly and is estimated to increase by 30% until 2030 (1.08 million new cases annually) (https://gco.iarc.fr/) (2, 3). The 5-year survival rate for patients with HNSCC is less than 50% before 2014 (4), primarily because approximately 80% to 90% of patients with advanced HNSCC develop local recurrence or distant metastasis (5, 6). Besides, patients with HNSCC have the second-highest suicide rate, less than patients with pancreatic cancer, which may be attributed to excessive stress and low life quality (7). Treatment strategies for HNSCC vary depending on the site of disease, disease stage, etc., and mainly include surgery (8), local radiotherapy (9), and systemic chemotherapy (10). Immunotherapy, especially immune checkpoint inhibitors, have also shown encouraging results in the advanced tumor (11, 12). However, for advanced squamous cell carcinoma, the currently existing therapies were of limited benefit.

Tumor microenvironment (TME) is the internal environment for the production and growth of tumor cells, which provides conditions for the initiation, proliferation, invasion, and metastasis of the tumor. It is closely related to the survival of tumor cells (13, 14). These immune cells and related stromal components, recruited and activated by tumor cells, form a tumor-suppressive inflammatory microenvironment in the early stages of tumor colonization or growth, thereby impeding tumor progression. However, after continuous tumor antigen stimulation and immune activation, the relevant effector cells in the microenvironment are in a state of depletion or remodeling. They cannot perform their normal functions or even promote tumors’ malignant manifestation, resulting in an immunosuppressive microenvironment (15). According to most previous studies, multiple immune cells and cytokines in the tumor microenvironment are closely associated with tumor prognosis (16). Currently, immunotherapy has achieved great success and long-term clinical benefits in treating various types of cancer. However, a significant proportion of patients still have limited or no response to immunotherapy. Strong immunosuppressive TME will cause tumor-reactive CD8+ T cells exhausted and lose their ability to eliminate cancer cells, thus reducing the effect of immunotherapy (15).

To investigate the TME status in patients with HNSCC and the underlying mechanism of prognostic influence, a series of analyses have been conducted around the relationship between TME and prognosis. In the present study, we used ESTIMATE software to analyze immune and stromal components’ scores in tumor tissues. The relationships between microenvironment and tumor prognosis in HNSCC were further explored through prognostic-related Cox regression analysis and TME-associated protein interaction networks (PPI) to search for predictive hub genes significantly associated with TME. CIBERSORT software was used to analyze the relative fraction of 22 immune cells in tumor tissues. The relationships between the expression of hub genes and the fraction of these tumor-infiltrating immune cells were also further analyzed.

## Materials and Methods

### Data Sources

The level 3 HTSeq-FPKM RNA-seq expression data and corresponding clinical data of HNSCC patients were retrieved from a data set of the Cancer Genome Atlas database (TCGA-HNSC) (https://portal.gdc.cancer.gov). A total of 546 RNA-seq expression data, including 502 tumor tissues and 44 normal tissues, were included in TCGA-HNSC data set, and were applied in the following analyses. A total of 499 patients had both clinical data containing survival information and gene expression data included for survival analysis (**Table S1**). GSE41613 data set retrieved from the Gene Expression Omnibus (GEO) database (http://www.ncbi.nlm.nih.gov/geo) with both expression data and the corresponding survival information of 97 patients were recruited for external survival validation. However, GSE41613 data set does not include gene expression data from normal tissues. Therefore, GSE30784 and GSE37991 data sets from the GEO database with the expression data of both tumor and normal tissues were then used for external RNA-seq expression validation. The GSE30784 data set includes gene expression data from 167 tumor tissues and 45 normal controls. A total of 80 tumor and non-tumor pair-wise samples obtained from 40 male oral squamous cell cancer (OSCC) patients were recruited in the GSE37991.

### Clinical Samples for Immunohistochemical Staining

To validate the target genes’ protein levels, we obtained a total of 10 pairs of primary tumor tissues and the corresponding adjacent normal tissues from OSCC patients who underwent surgical resection at the Department of Stomatology, Chinese PLA General Hospital, between March 2020 and July 2020. The location of tumor occurrence in 10 patients included six cases of the mouth’s fundus, 4 cases of the gingiva. There were seven cases in males and three cases in females. All patients had postoperative pathology confirmed as oral squamous cell carcinoma. All patients underwent surgery prior to chemotherapy or radiotherapy. All samples were used for immunohistochemical staining. Our study was conducted following the Declaration of Helsinki and approved by the Chinese PLA General Hospital’s Ethics Committee. All patients signed an informed consent form.

### Scores for TME Using ESTIMATE Package

The ratio of immune and stromal cells in the TME of each sample was calculated using the ESTIMATE package (Version 1.0.13) based on the R software version 3.6.1. The data used were HTSeq-FPKM RNA-seq expression data. Results are presented in the form of three scores, including ImmuneScore, StromalScore, and ESTIMATEScore.The higher the respective score, the greater the ratios of the corresponding components in the TME. If the overall score is higher, the proportion of tumor cells in the tumor tissue is lower with lower tumor purity.

### DEGs Between High- and Low-Score Groups Regarding ImmuneScore and StromalScore

The 502 tumor samples were labeled as high scores or low scores based on the median value of ImmuneScore or StromalScore, respectively. DEGs were obtained by performing differential analysis of gene expression between groups using the “limma” package based on R software. DEGs with a log2(folder change) >1 and a false discovery rate (FDR) adjusted *P*-value < 0.05 were considered statistically significant. Genes that were differentially expressed in both immune and stromal components were included in the next step of the analysis. A total of 275 DEGs associated with changes in the TME were included in the next stage of analysis after matching.

### Survival Analysis

Survival analysis was performed by applying the R software package “survminer” (Version 0.4.7) and “survival” (Version 2.44.1.1). Patients included in the survival analysis must have both gene expression data and corresponding survival clinical data. A total of 499 individuals were eventually included in the survival analysis. Tumors were categorized into high and low scoring groups based on the above microenvironmental scores, and the survival differences between the groups were compared. Also, patients were grouped according to their gender, followed by survival analysis. Survival curves were plotted using the Kaplan-Meier method. A *P* value < 0.05 of the log-rank test was considered statistically significant.

### Gene Ontology (GO) and The Kyoto Encyclopedia of Genes and Genomes (KEGG) Pathway Functional Enrichment Analysis

GO and KEGG enrichment analysis was performed utilizing DEGs. We completed the GO and KEGG pathway analysis using R packages (“enrichplot,” “clusterProfiler,” and “ggplot2”) (17). We used both *P*-value < 0.05 and FDR adjusted *P*-value < 0.05 as the threshold for GO and KEGG enrichment analysis.

### Protein-Protein Interaction (PPI) Network of DEGs and Hub Genes

DEGs that are significantly associated with TME were used to construct the PPI network to analyze the model’s intrinsic function. The PPI network construction was based on the STRING database (https://string-db.org/), and we subsequently applied Cytoscape software (version:3.8.0) for the reconstruction and visualization of the network. Nodes with the confidence of an interactive relationship > 0.7 were used for building the network.

After constructing the PPI, the core genes of the network need to be identified. We counted the number of neighboring nodes for each gene and selected the top 30 genes with the most neighboring nodes as the PPI network’s core genes.

### Prognosis-Related Genes in DEGs

First, Univariate Cox regression analysis was used to identify the genes associated with prognosis. A forest plot was constructed based on the Cox analysis. *P <*0.05 indicated a significant association with prognosis. Hazard Ratio (HR) >1 indicated a high-risk gene. Kaplan–Meier test was also used. Patients were divided into two groups with high and low expression according to the median value of gene expression to compare whether there was a difference in survival between them. The genes that were significant by both methods were used as the final prognosis-related genes.

### Gene Set Enrichment Analysis (GSEA)

GSEA is used to assess genes’ distribution trends in a predefined set of genes in a gene set sequenced for phenotypic relevance and determine their contribution to the phenotype (18). We applied the GSEA software (Version 4.0.3, http://software.broadinstitute.org/gsea/) with 1,000 phenotype permutations for GSEA enrichment analysis. The statistically significant gene sets threshold was set to Nominal *P*-value < 0.05 with an FDR adjusted *P*-value < 0.25. We classified the patients into two groups according to the median value of a target gene. We then performed a GESA analysis to compare the pathways of differential enrichment between the two groups. MSigDB oncogenic signatures gene sets (version 7.1, https://www.gsea-msigdb.org/gsea/downloads.jsp) was applied in the GSEA analysis.

### Estimation of Immune Cell Abundance in Tumor Tissue

To analyze whether there were differences in the immune cell abundance of tumor tissue between High and low expression groups of hub genes, we used CIBERSORT (https://cibersort.stanford.edu/) to evaluate the relative abundance of predefined cell types in mixed solid tissues. The data used were normalized FPKM gene expression data of tumor tissue. We used the default LM22 leukocyte gene signature matrix from the CIBERSORT website. LM22 contains 547 genes distinguishing 22 types of immune-related cells. Disabling quantile normalization was checked. We set the number of permutations to 1000 for robust analyses. CIBERSORT enumerated the relative proportions of the 22 infiltrating immune cells, including B cells, dendritic cells, T cells, natural killer cells, macrophages, and others.

### Differential Expression Analysis of Target Genes

The Wilcox rank-sum test was used to compare the differences in the expression of target genes between tumor and normal tissues. The R package “beeswarm” was used to generate the corresponding expression figures. Besides, we then performed paired differentiation analyses of target gene expression in tumor and normal samples from the same patient using the Wilcoxon rank-sum test. Statistical thresholds were set at *P*<0.05. The figures were generated using the “ggpaired” command of the R package “ggpubr.”

### Data Sets Validations of the Hub Genes

The relationships between hub genes and prognosis were analyzed in TCGA-HNSC and were validated using the GSE41613 external data set (n = 97) and two randomly generated subsets of TCGA-HNSC (n = 250 and n = 249). Another two external data sets (GSE30784 (n = 212) and GSE37991 (n = 80)) were used to validate the expression of hub genes (CCR4, CCR8, and P2RY14) between normal and tumor tissues. To validate the gender differences, we then divided TCGA-HNSC data set into two sub-data sets by gender (male = 366, female = 133).

### Correlation of Target Gene Expression With Clinicopathological Characteristics

The Kruskal-Wallis rank-sum test was used to compare statistical differences between multiple groups. Wilcoxon rank-sum test was used to perform statistical analysis for comparison between two groups. *P <*0.05 was considered statistically significant. Subsequently, both univariate and multivariate Cox proportional hazards regression analyses of the hub genes and other clinical factors for the overall survival (OS) time were performed in the TCGA cohort.

### Immunohistochemical Staining

Tumor tissues and control tissues were fixed with formalin, dehydrated, paraffin-embedded, and cut into 5-μm-thick continuous sections. After dewaxing and hydration, the sections were infiltrated with closed permeabilization solution for 30 min, washed three times with 0.01 mol/L PBS for 3 min each time, blocked with 5% BSA for 1 h, and treated with anti-*CCR8* antibody (ZENBIO, No. 860154) or anti-*P2RY14* antibody (Bioss, Bs-15356R) (diluted to 1:250) in 4°C overnight incubation. After a brief wash with PBS, the antibody was incubated with a horseradish peroxidase-conjugated anti-rabbit IgG antibody (1:500) for 2 h, and the staining was then developed with 0.003% H_2_O_2_ and 0.03% 3,3′-diaminobenzidine in 0.05 mol/L Tris-HCl. The staining was observed, the dye was poured off, and the color development time was controlled for about 3 to 10 min. The neutral resin was used to seal the slices and then observed and photographed under the microscope. For the quantification, 10 non-overlapping cortical section at a magnification of 400× (high power field, HPF) were counted using Image-pro plus software. Multiple t-test was used to evaluate the difference between tumor and normal samples. The statistical threshold was set at *P*<0.05.

## Results

**Figure 1:**
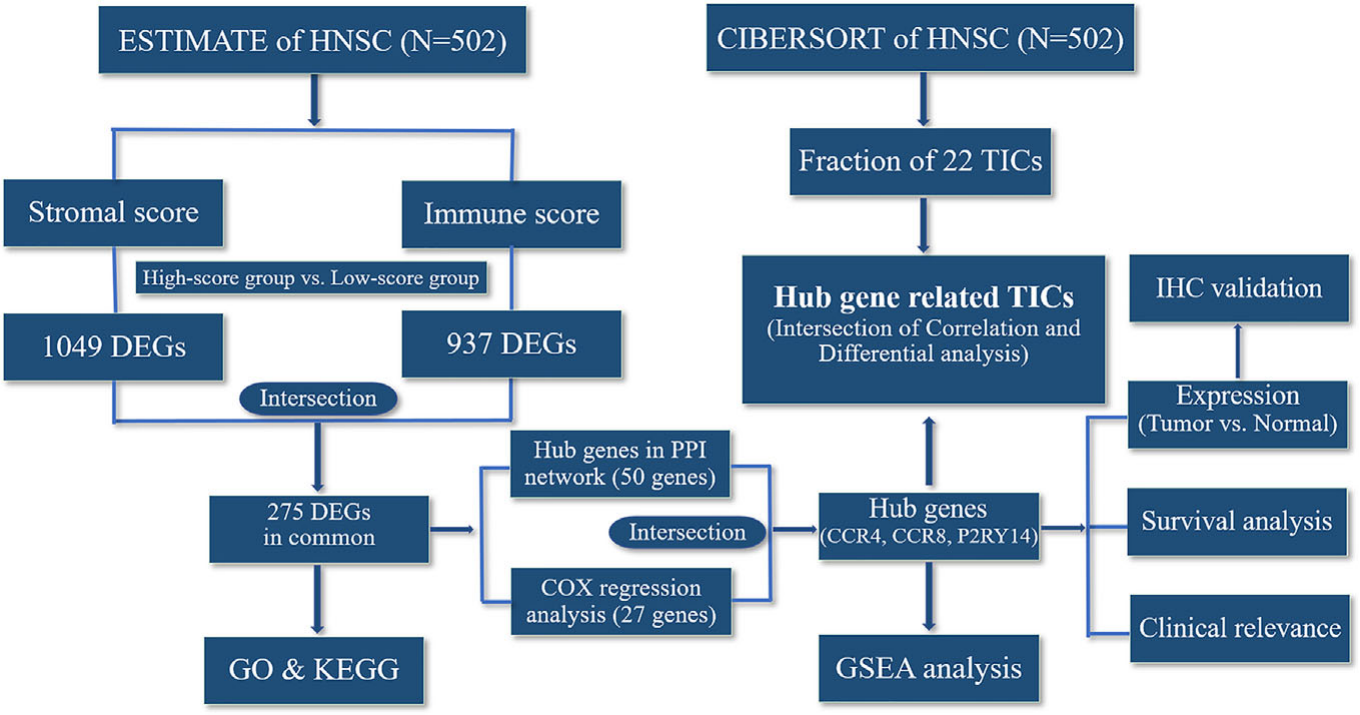
Flowchart of the entire analysis.

The flowchart for the entire analysis is shown in **Figure 1**.

### Clinical Relevance of TME Scores

**Figure 2:**
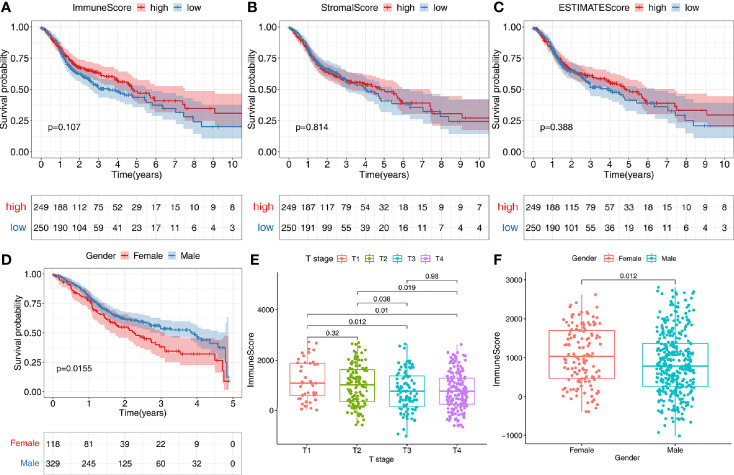
Correlation of tumor microenvironment (TME) with survival and clinical characteristics. **(A–C)** According to the median value of the immune score, stromal score, or estimate score, 502 patients were stratified into the high- or low-score group. Kaplan-Meier curves were used for survival analyses between different score groups in the TCGA-HNSC data set. **(D)** Females have a worse prognosis than male patients with an OS<5 years (*P*<0.05). **(E)** There was a significant correlation between immune score and T stage (*P*<0.05). **(F)** Immune scores of tumor tissues were significantly lower in male patients than in female patients (*P*<0.05).

According to the median value of the TME scores, respectively, 502 patients in TCGA-HNSC data set were stratified into the high- or low-score group. All TME scores analyses did not find a significant correlation with survival (*P* > 0.05) (**Figures 2A–C**). However, we can observe a tendency that patients with higher immune scores have a better prognosis (**Figure 2A**). In addition, we found that female patients had a significantly worse prognosis than male patients (*P* < 0.05) (**Figure 2D**). There was a significant correlation between immune score and T stage (*P* < 0.05) (**Figure 2E**). Immune scores of tumor tissues were significantly lower in male patients than in female patients (*P* < 0.05) (**Figure 2F**). No correlation between tumor immune score and lymph node metastasis in patients was found (**Figure S1A**). No significant correlation between tumor StromalScore and any clinical features was found in TCGA-HNSC data set (*P* > 0.05) (**Figures S1B–D**).

### DEGs Between High- and Low-Score Groups Regarding ImmuneScore and StromalScore

**Figure 3:**
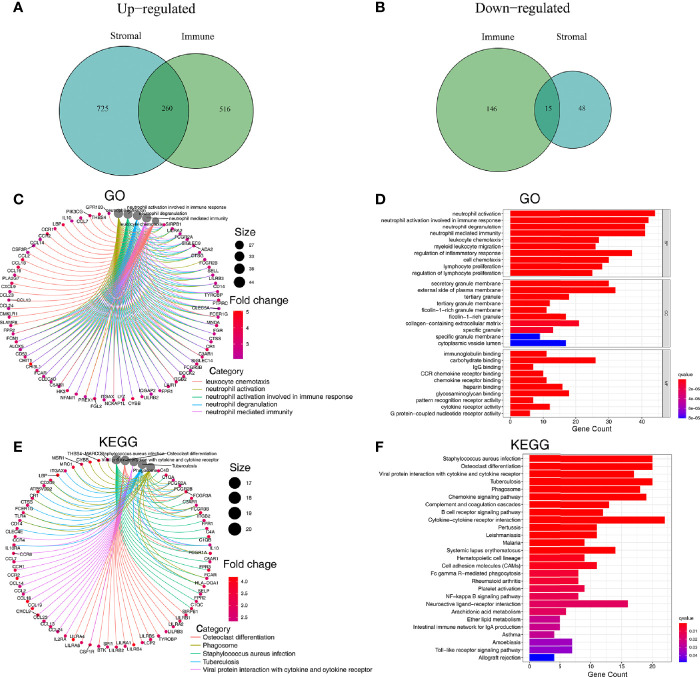
Analysis of genes affecting the TME and their associated GO and KEGG enrichment. **(A, B)** Venn plots showing 260 up-regulated DEGs and 15 down-regulated DEGs shared by ImmuneScore and StromalScore (thresholds for differential gene expression set at an FDR adjusted *P*-value <0.05 and log2FC>1). **(C, D)** GO enrichment analysis for 275 microenvironmentally relevant DEGs, the statistical threshold set at *P*-value < 0.05 with an FDR adjusted *P*-value < 0.05. **(E, F)** KEGG enrichment analysis for 275 microenvironmentally relevant DEGs, the statistical threshold set at *P* value < 0.05 with an FDR adjusted *P*-value < 0.05.

The 502 tumor samples were labeled as a high-score or low-score based on the median value of ImmuneScore or StromalScore, respectively. Regarding ImmuneScore, 776 genes were up-regulated, and 161 genes were down-regulated in the high-score group compared to the low-score group. For the stromal component scores, 985 genes were up-regulated, and 63 genes were down-regulated in the high-score group compared to the low-score group. There were 275 genes whose expression was simultaneously and significantly correlated with either the immune component or the stromal component (**Figures 3A, B**).

### GO and KEGG Pathway Analyses of the DEGs

Since these 275 DEGs were significantly associated with TME status, we sought to explore these genes’ function through GO and KEGG enrichment analysis. Results of GO enrichment analysis revealed that DEGs were enriched significantly in neutrophil activation, neutrophil degranulation, neutrophil-mediated immunity, leukocyte chemotaxis, regulation of inflammatory response, lymphocyte proliferation (*P*<0.05 and FDR adjusted *P*<0.05) (**Figures 3C, D**, **Figure S2A**). Results of KEGG functional enrichment analysis revealed that DEGs were significantly enriched in Cytokine−cytokine receptor interaction, Chemokine signaling pathway, Staphylococcus aureus infection, and others (*P* < 0.05 and FDR adjusted *P*<0.05) (**Figures 3E, F**, **Figure S2B**).

### Prognosis-Related Genes in DEGs

**Figure 4:**
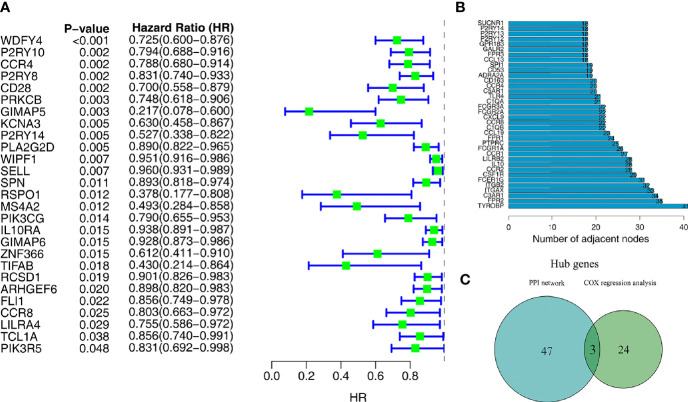
Screening of target genes by PPI network and survival analysis. **(A)** Univariate Cox regression analysis of 275 microenvironment-associated DEGs. **(B)** Core genes with the highest number of neighboring nodes in the PPI network. **(C)** The Wayne plot shows three hub genes that belong to both the PPI network and prognosis-related genes.

Univariate Cox regression analysis and the Kaplan–Meier method were both used to identify the genes associated with prognosis. A total of 27 genes were found to be significantly associated with prognosis (*P*<0.05 in both Cox regression analysis and Kaplan–Meier analysis) (**Figure 4A**).

### PPI Network and Hub Genes

With the 275 DEGs, the PPI network construction was based on the STRING database, and we subsequently applied Cytoscape software for the reconstruction and visualization of the network. A total of 182 network nodes with the confidence of an interactive relationship larger than 0.7 existed in the PPI network. We counted the number of neighboring nodes for each gene and selected the top 30 genes with the most neighboring nodes as the hub genes of the PPI network (**Figure 4B**).

### Hub Genes Related to TME and Prognosis

To further explore the relationship between TME and prognosis, the intersection analysis between the critical nodes in the PPI network and the prognosis related DEGs was carried out, and only three genes, *CCR4*, *CCR8*, and *P2RY14*, were overlapping from the above analyses (**Figure 4C**).

### Differential Expression of Key Genes Between Tumor and Normal Tissues

**Figure 5:**
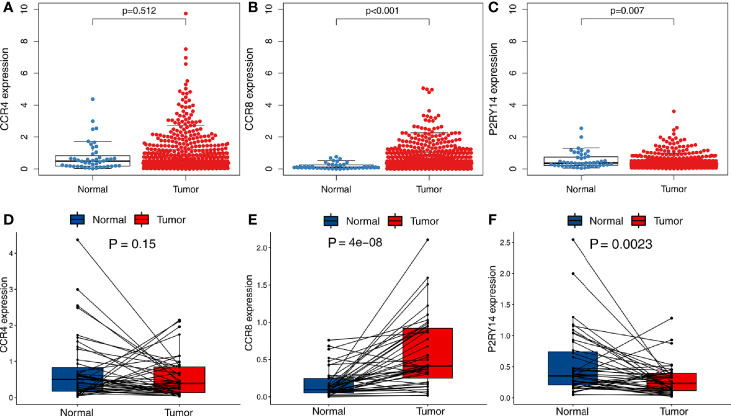
Differential expression analysis of hub genes in tumor and normal tissues in TCGA-HNSC data set. **(A)** There was no significant difference in the expression of *CCR4* in tumor and normal tissues. **(B, C)** Significant differences were observed in the expression of *CCR8* and *P2RY14* in tumor and normal tissues (*P*<0.01). **(D–F)** A paired analysis of tumor and normal tissues from the same patients confirmed the previous analysis results.

A total of 502 tumor tissues and 44 normal tissues were included in TCGA-HNSC data set. We used the “limma” package to analyze the expression of these three key genes and found that *CCR8* and *P2RY14* were differentially expressed in tumor tissues compared with normal tissues (*P*<0.01), while *CCR4* exhibited no significant difference in expression between tumor and normal tissues (**Figures 5A–C**). Paired differentiation analyses using the Wilcoxon rank-sum test exhibited similar results. A total of 43 pairs of tumors and normal tissues from 43 patients were included in this analysis. *CCR8* and *P2RY14* were significantly differentially expressed in tumor tissues compared with normal tissues, while *CCR4* was not significantly different (**Figures 5D–F**).

**Figure 6:**
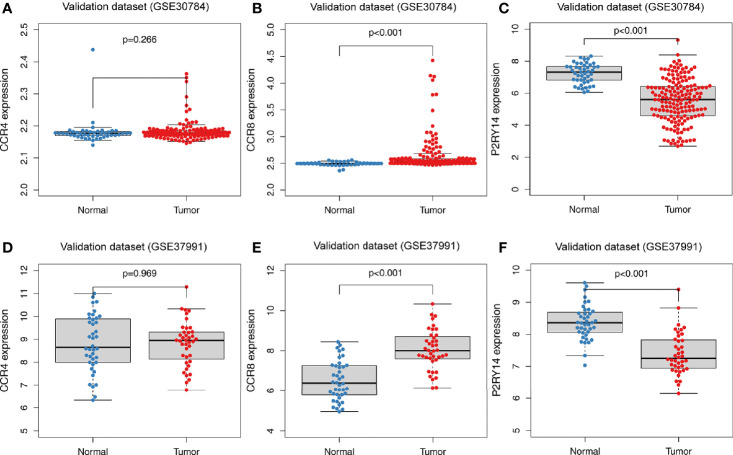
Differential expression analysis of hub genes in tumor and normal tissues in GEO data sets. **(A)** There was no significant difference in the expression of *CCR4* in the GSE30784 data set. **(B, C)** Significant differences were observed in the expression of *CCR8* and *P2RY14* between tumor and normal tissues in the GSE30784 data set (*P*<0.001). **(D)** No significant difference in *CCR4* was observed in the GSE37991 data set. **(E, F)** In the GSE37991 data set, *CCR8* expression was increased in tumor tissues compared to normal tissues, while *P2RY14* expression was significantly decreased.

GSE30784 and GSE37991 were used to validate the above-mentioned expression results of hub genes. According to the results from TCGA-HNSC data set, compared to normal tissues, *CCR4* exhibited a similar expression level in the tumor tissues (*P*>0.05) (**Figures 6A, D**). The expression of *CCR8* was significantly increased in tumor tissues (*P*<0.001) (**Figures 6B, E**), while the expression of *P2RY14* was significantly decreased (*P*<0.001) (**Figures 6C, F**).

To validate the gender differences, we then divided TCGA-HNSC data set into two groups by gender. We found no significant correlation between higher or lower immune scores and tumor prognosis in either the male or female groups (*P*>0.05) (**Figures S3A, B**). However, in the male group, there was a trend for better prognosis in the higher immune score group (**Figure S3A**). Within these two groups, respectively, we performed survival analysis based on the high and low expression of the hub genes. Interestingly, in the male group, the expression of all genes was significantly correlated with survival (P<0.005), while in the female group, the expression of all hub genes was not correlated with survival (P>0.05) (**Figures S3C–H**).

**Figure 7:**
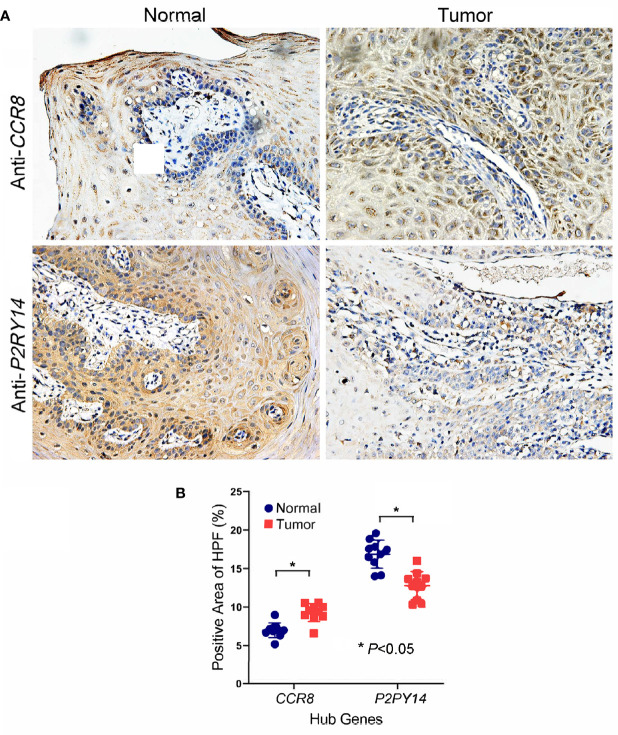
Immunohistochemical analysis of oral squamous cell carcinoma specimens and adjacent normal tissues for *CCR8* and *P2RY14* protein. **(A)** *CCR8* was highly expressed in tumor specimens of HNSCC patients compared to normal tissues. *P2RY14* was highly expressed in normal tissues compared to tumor specimens. **(B)** The quantification of IHC results (FDR adjusted P-value < 0.05).

Immunohistochemical analysis of HNSCC specimens and adjacent normal tissues for *CCR8* and *P2RY14* proteins revealed that *CCR8* was significantly over-expressed in HNSCC specimens, whereas *P2RY14* was significantly lower expressed than in normal tissues (*P*<0.05) (**Figures 7A, B**).

### The Correlation of Gene Expression With the Survival and Clinical Characteristics

**Figure 8:**
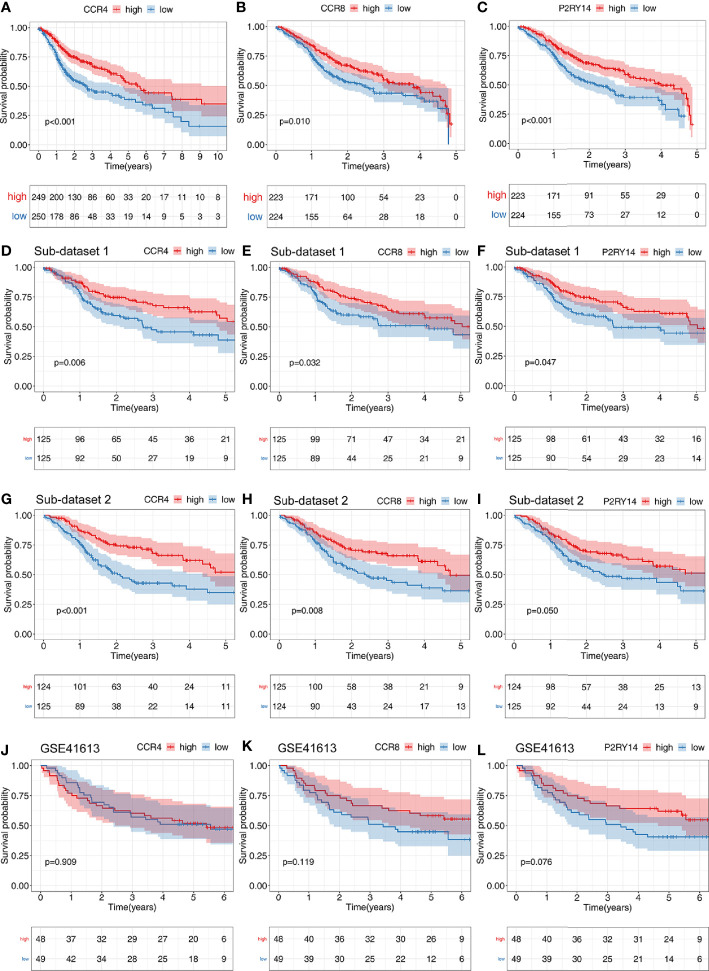
Results of survival analyses of hub genes. The expression of *CCR4* **(A)**, *CCR8* **(B)**, and *P2RY14* **(C)** were all significantly correlated with patient prognosis in the TCGA data set, and the higher the level of gene expression, the better the patient’s prognosis. **(D–I)** Survival analysis of two randomly distributed subsets showed results consistent with the original data set, with the expression of all of the three hub genes significantly correlated with survival. **(J–L)** Survival analysis was performed on another validation data set GSE41613.

*CCR4* showed a significant correlation with survival in TCGA-HNSC data set (*P*<0.001), and the higher the gene expression, the better the patient’s prognosis (**Figure 8A**). To eliminate the non-proportional hazard, according to the survival period of the patients, we classified those with less than 5 years as a group and those with more than 5 years were included in another group for separate analysis of *CCR8* and *P2RY14*. We found the vast majority (447/499) of patients with a survival of less than 5 years. The results of the survival analysis showed that the expression of *CCR8*, *P2RY14* were significantly associated with survival in patients with OS less than 5 years (**Figures 8B, C**). However, the results were not significant in patients with OS more than 5 years (n=52) (**Figures S4A, B**).

In the two randomly generated sub-data sets of TCGA-HNSC, we obtained results from the survival analysis that were highly consistent with previous results, with higher expression of the hub genes predicting a better prognosis (*P*<0.05) (**Figures 8D–I**).

We also validated the results on the external GSE41613 data set and found that no significant differences were found for any of the three genes, but CCR8 and P2RY14 showed highly identical trends to those in TCGA-HNSC (**Figures 8J–L**).

**Figure 9:**
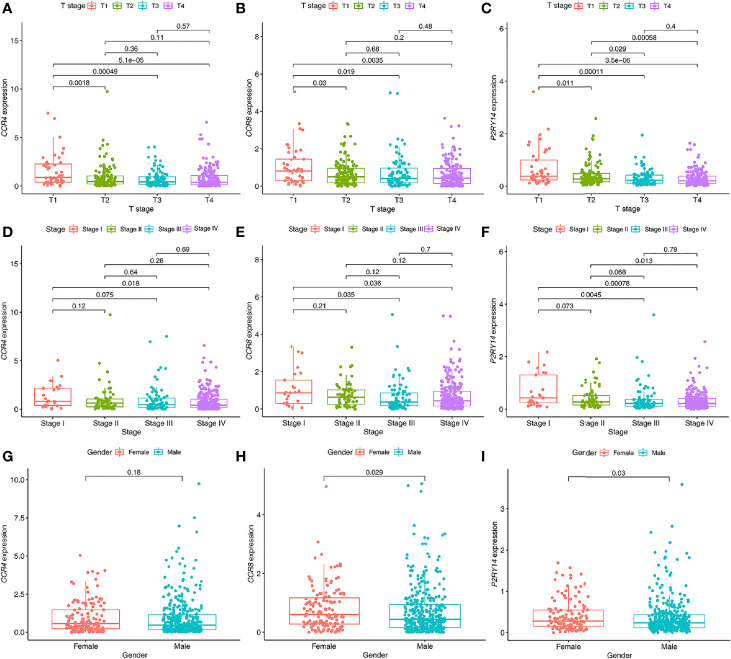
Relationship between the expression levels of hub genes in tumor tissues and clinical characteristics of patients. **(A–C)** The expression of *CCR4* **(A)**, *CCR8* **(B)**, and *P2RY14* **(C)** were all significantly correlated with the T staging. The expression levels of these genes all decreased gradually with the progression of the T stage. **(D–F)** Similar results were seen with the pathological staging in all three hub genes, but not as significant as for T staging. **(G–I)** All of these genes were less expressed in males than in females, with no significant difference was shown in CCR4 (*P*>0.05), and significant differences were demonstrated in both *CCR8* and *P2RY14* (*P*<0.05).

*CCR4* gene expression was significantly correlated with pathological tumor stage and T-stage (*P*<0.05) (**Figure 8**). Both *CCR8* and *P2RY14* gene expression was significantly correlated with pathological tumor stage, T-stage, and gender (*P*<0.05) (**Figure 9**).

**Figure 10:**
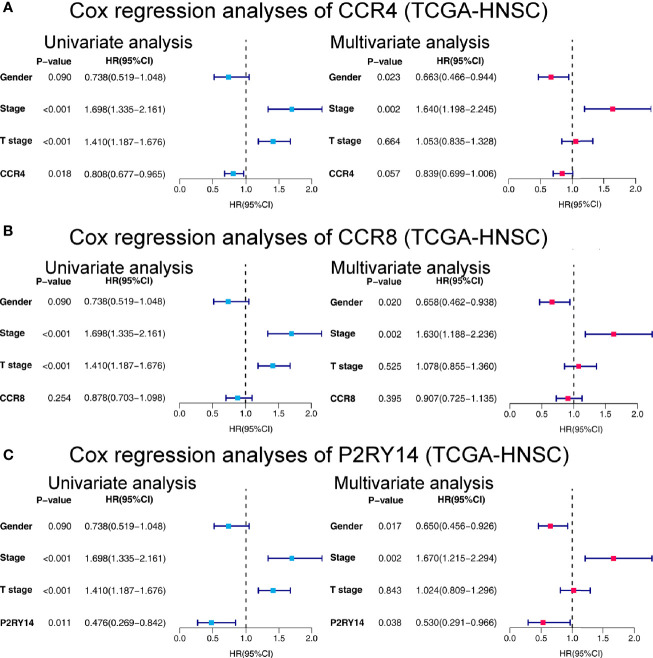
Forest plots of univariate and multivariate Cox regression analyses in TCGA-HNSC. **(A)** Cox regression analyses on the relationship between CCR4 expression and prognosis. **(B)** Cox regression analyses of CCR8. **(C)** Cox regression analyses of P2RY14. HR, hazard ratio.

CCR4 demonstrated its independent predictive value of OS in the univariate Cox regression analysis in TCGA-HNSC data set (*P*<0.05). In the multivariate regression analysis of CCR4, although not significant, it showed a predictive value that was not weaker than that of the T-stage (*P* = 0.057) (**Figure 10A**). CCR8 expression did not reveal its independent prognostic value over other factors in the regression analyses (**Figure 10B**). Notably, compared to gender and stage, we found the expression of P2RY14 exhibits an independent prognostic value in both the univariate and multivariate Cox regression analyses (*P <*0.05) (see in **Figure 10C**).

### Pathways Enriched for These Hub Genes as Revealed by GSEA Analysis

**Figure 11:**
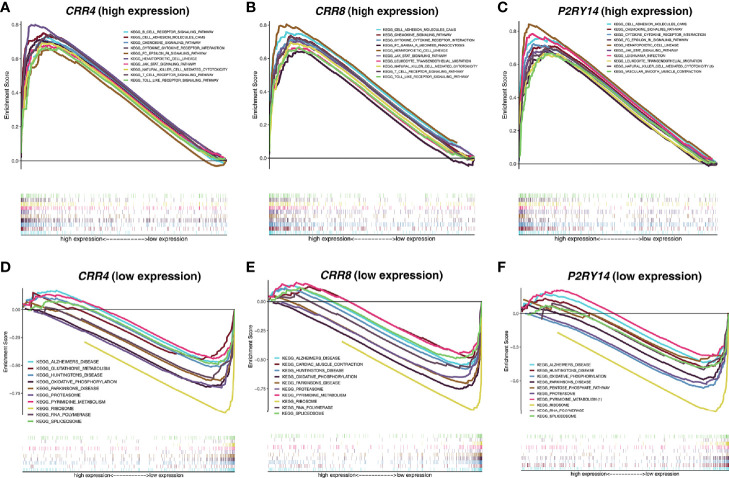
Functional enrichment analyses regarding the expression of the hub genes. **(A–C)** Results of enrichment analysis with higher expression of the hub genes. **(D–F)** Results of enrichment analysis with lower expression of the hub genes. The threshold was set to Nominal *P*-value < 0.05 with an FDR adjusted *P*-value < 0.25.

Given that the expression levels of all three key genes mentioned above were significantly correlated with the survival and T stage of patients in TCGA-HNSC data set, we categorized the patients into high and low expression groups according to their median expression levels and then performed GSEA analysis. The results showed that in the high-expression group, all three hub genes were significantly enriched in immune-related pathways such as “CYTOKINE_CYTOKINE_RECEPTOR_INTERACTION,” “JAK_STAT_SIGNALING_PATHWAY,” “CHEMOKINE_SIGNALING_PATHWAY,” “NATURAL_KILLER_CELL_MEDIATED_CYTOTOXICITY” (**Figures 11A–C**). In the low-expression group, all three genes are significantly enriched in metabolism-related pathways, including “OXIDATIVE_PHOSPHORYLATION,” “RNA_POLYMERASE,” “RIBOSOME,” and so on (**Figures 11D–F**).

### Correlation Between Gene Expression and Immune Cell Infiltration

**Figure 12:**
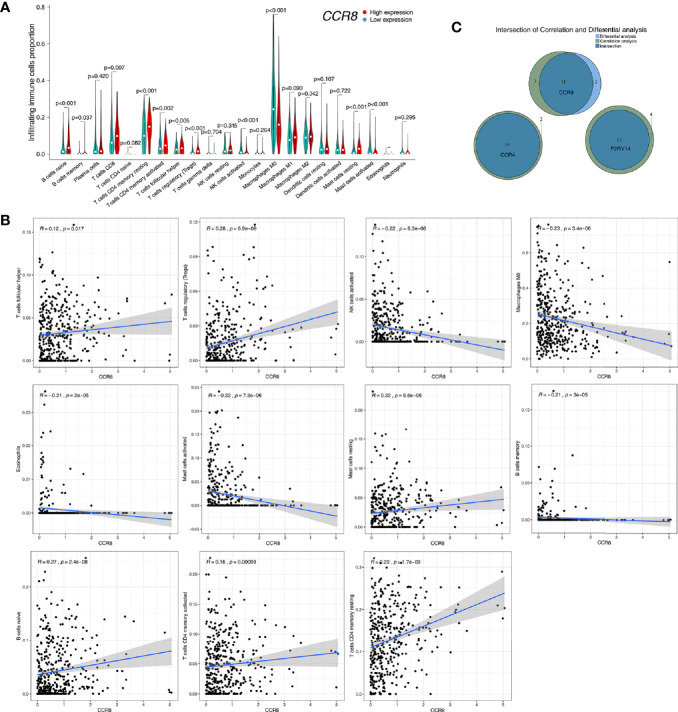
Correlation of *CCR8* expression with immune cell fraction in tumor tissues in TCGA-HNSC. **(A)** Patients were divided into high- and low-expression groups based on the median value of *CCR8* gene expression. There were significant differences in the fraction of some immune cells in the tumor tissue (*P*<0.05). **(B)** Correlation analysis of *CCR8* and immune cell content was used to validate the above differential analysis results. **(C)** Wayne plots showed that the expression of *CCR4*, *CCR8*, and *P2RY14* was associated with the fraction of several immune cells, respectively.

CIBERSORT was used to estimate the fractions of 22 infiltrated immune cells using the RNA-sequence data. The relative abundance of parts of the 22 infiltrated immune cells exhibited significant differences between the high- and low-expression groups. Combining the results of differential analysis (**Figure 12A**, **Figure S5A**, **Figure S6A**) and correlation analysis (**Figure 12B**, **Figure S5B**, **Figure S6B**), we found that the content of a total of 14 immune cells was significantly correlated with the expression of *CCR4*, 11 with the expression of *CCR8*, and 13 with the expression of *P2RY14* (**Figure 12C**). For example, six immune cells were positively correlated with the expression of *CCR8*, including naive B cells, T cells regulatory (Tregs), T cells CD4 memory resting, T cells follicular helper, resting Mast cells, and activated T cells CD4 memory. Five cells were negatively correlated with *CCR8* expression, including memory B cells, Macrophages M0, activated Mast cells, Eosinophils, and activated NK cells. Notably, five of the infiltrating cells had similar correlations with all three hub genes. The expression of these genes was negatively correlated with the fraction of memory B cells and positively correlated with the fraction of the other four cells, including naive B cells, resting T cells CD4 memory, T cells follicular helper, and Tregs. These results further support that the expression of *CCR4*, *CCR8*, and *P2RY14* affect the immunoreactivity of TME.

## Discussion

In this study, we identified three hub genes (*CCR4*, *CCR8*, and *P2RY14*) that were significantly associated with both TME and prognosis through a combination of correlation analysis and survival analysis for the TME related DEGs. Among them, there were significant differences in the expression of *CCR8* and *P2RY14* in tumor tissues and normal tissues. Enrichment analysis revealed that the functions of these genes are primarily associated with the immune system. In a subsequent analysis of tumor-infiltrating cells, we found that the expression of these genes was all significantly correlated with the fraction of multiple immune cells in the tumor tissue. This is further evidence that these genes influence the immune system and the tumor microenvironment by influencing the immune system and, thus, tumorigenesis, progression, and prognosis. They can also be used as reliable predictors of the tumor microenvironment and prognosis.

A great number of studies have confirmed that TME plays an essential role in tumor initiation and development (19–21). An aggressive TME may support continued tumor growth, invasion, and metastasis. The interactions between numerous components of TME may result in different tumor growth patterns and prognosis, but the precise mechanisms remain to be elucidated and thoroughly investigated. Li et al. recently found that three immune-related genes (*COL1A1*, *COMP*, and *SERPINE2*) are significantly correlated in both TME and tumor prognosis, thus revealing the possible effects and pathways of microenvironment on tumor prognosis (22). Huo et al. analyzed the scores of 22 immune cells in tumor tissues using gene expression data from 816 HNSCC patients and developed a TME score-based prognostic risk model accordingly. The model was significantly correlated with tumor stage, grade, HPV infection, and prognosis (23).

Both *CCR4* and *CCR8* belong to chemokine receptors, and the proteins encoded by these genes belong to the family of G-protein-coupled receptors (24). Chemokines consist of a set of small polypeptides that regulate cellular transport in various types of leukocytes. Chemokines also have essential roles in the development, homeostasis, and function of the immune system, and they have effects on endothelial cells involved in angiogenesis or vasorelaxation (25). Besides, these genes are also strongly associated with tumor development. Xu et al. found that *CCR4* and CCR6 are independent prognostic indicators for patients with lung adenocarcinoma (26). One animal study in canines confirmed that the expression of *CCR4* was significantly higher in tumor tissues than in normal tissues and was significantly correlated with the content of Treg cells. They suggest that the *CCL17*/*CCR4* axis may drive Treg recruitment in a variety of canine cancers. *CCR4* blockade may be a potential therapeutic option to eradicate tumors through Treg depletion (27). Based on the mechanism of *CCR4*, Mogamulizumab is a new humanized anti-*CCR4* antibody that is currently being used in the clinical treatment of advanced cutaneous T-cell lymphoma (CTCL) (28).

*P2RY14* is a purinergic receptor for UDP-glucose and other UDP-glycans coupled to G-proteins. It plays a role in the immune system by participating in the regulation of the stem cell compartment (29). *P2RY14* is now thought to be closely associated with tumorigenesis and development. For instance, Wang et al. found that *P2RY14* is significantly down-regulated in lung cancer tissues based on analysis of different data sets, and the higher the expression level of this gene, the better the patient’s prognosis (30). Similar to the results we obtained in TCGA-HNSC data set, the higher the expression of *P2RY14* in the tumor tissue, the better the prognosis of the patient (**Figure 8C**). And in tumor tissues, the levels of the gene are significantly down-regulated compared to normal tissues (**Figures 5C, F**). Immunohistochemical analysis also validated the results of these genetic analyses.

In addition, we analyzed the correlation between these hub genes and clinical features, and several meaningful results were obtained. The first was that the expression of these genes was significantly correlated with the T-stage, with differences in the expression of the genes in the different stages. In all three genes, this significance was observed, and the difference in their expression between T1 and all other stages became increasingly significant with increasing staging. We also observed such differences in pathological staging. This supports that the expression of these genes was significantly correlated with tumor progression. Interestingly, the expression levels of *CCR8* and *P2RY14* in tumor tissues were significantly correlated with gender, with women showing significantly higher levels of gene expression than men. The expression of *CCR4*, although non-significant, showed a similar tendency. However, these findings have not been reported in previous studies. Notably, a similar result was seen in a previous analysis of TME scores, with male patients having significantly lower immune scores than females. Related studies have found significant differences in incidence, outcome, and response to immunotherapy across tumors (31, 32). For example, in urothelial bladder carcinoma, although the incidence was lower in women than in men, the disease’s aggressiveness and mortality were higher in women than in men (33). Similar to these studies, we grouped the data by gender in this study and found that female patients had a poorer prognosis than male patients (*P* < 0.05, HR= 1.328 (0.981 to 1.799)) (**Figure 2D**). Although we found that immune scores were higher in women than in men, the results showed that the prognosis of female patients was not better than men as expected from the immune scores. Therefore, it is reasonable to assume that although females have more immune infiltrates and highly expressed hub genes than males, this is not sufficient to have a decisive impact on the survival prognosis. The reasons for this may be related firstly to the smaller sample size of women and more likely, to the fact that female HNSCC patients differ significantly from men at the molecular level (34). These results suggest that there may be differences in TME between male and female patients. No significant gender-related pathways were also found in the results of the KEGG enrichment analysis. We may need to do more gender-related studies to reveal the possible underlying mechanisms.

The relative fraction of tumor infiltrated immune cells are estimated by Cibersort software using the FPKM RNA-seq expression data. By correlation and differential analyses, we found that the content of these immune-related cells in tumor tissues was closely associated with the three hub genes’ expression. We found that both in *CCR4*, *CCR8*, and *P2RY14*, the abundance of CD8 T-lymphocytes was significantly higher in the group with high expression of these genes than in the lower expression group. T cells expressing CD8 usually differentiate into cytotoxic T-lymphocytes after being activated and are able to kill tumor cells specifically. Therefore, studies have confirmed that the higher the level of CD8+ T cells, the stronger the body’s anti-tumor immune response and the better the prognosis of patients (35, 36). In all of the three hub genes, we also found that the content of M0 macrophages was significantly lower in the high-expression group of the gene. The primary function of macrophages is phagocytosis and digestion of cellular debris and pathogens, and activation of other immune cells. M0 macrophages are non-activated macrophages without any inflammatory or tumor-associated function and can be transformed into classically activated M1 macrophages and alternatively activated M2 macrophages (37, 38). M1 macrophages have a primary anti-tumor role, distinguishing tumor cells from healthy cells, recognizing and then killing tumor cells by mediating cytotoxic effects. In contrast, the role of M2 macrophages is to promote the growth and metastasis of tumors. In this study, we found that the content of M0 macrophages was significantly reduced in the high expression group while M1 macrophages were increased although there was no significant difference, while there was no significant change in M2 macrophages, which suggested an enhanced anti-tumor immune response in the high expression group. These results are also consistent with the changes in immune status caused by altered CD8 T cells. Furthermore, in the present study, we found that, like the other two key genes, high expression of the *CCR8* gene was significantly associated with an enrichment of Tregs in tumor tissue, which in turn predicted a better prognosis. This is consistent with previous findings in colorectal cancer, which suggested that enrichment of Tregs in tumors favors a better prognosis (39, 40). Salama et al. found that improved survival associated with a high density of tumor-infiltrating FOXP3(+) Tregs in colorectal cancer (40). These results confirm the influence of hub genes on different types of tumor immune cells and also preliminarily reveal possible mechanisms that may influence the microenvironment and prognosis.

There are several limitations to this study. Firstly, this study calculated the TME score of tumor tissues based on mRNA expression data using the ESTIMATE algorithm and the fraction of the immune cells using the CIBERSORT algorithm. These algorithms are still in the exploratory stage. More optimization of the algorithms is needed in the future to be closer to the real world. Second, we found three hub genes, but it is unclear through what functions and pathways these genes affect TME and prognosis, and further studies are needed in the future. In addition, we included a limited number of patients for IHC validation. The sample size should be enlarged in future studies.

## Conclusion

In the present study, we identified three hub genes (*CCR4*, *CCR8*, and *P2RY14*) that are significantly associated with TME and prognosis. We found that these genes are essential in building a microenvironment that stimulates anti-tumor immune responses and thus inhibits tumor growth and migration. These findings may shed new light on future anti-tumor therapies that target TME status.

## Data Availability Statement

The data sets presented in this study can be found in online repositories. The names of the repository/repositories and accession number(s) can be found in the article/**Supplementary Material**.

## Ethics Statement

The studies involving human participants were reviewed and approved by the Ethics Committee of Chinese PLA General Hosptial. The patients/participants provided their written informed consent to participate in this study.

## Author Contributions

LM and YX conceived and designed the study. BQ was responsible for patients’ samples collection. QH and JL helped to conduct some experiments including IHC staining. LM and XH performed the data analyses. XBZ and BW validated the results. LM drafted the manuscript with YW. YX, ZY and XZ reviewed and revised the manuscript. ZY guided the article revision and the selection of statistical methods. All authors contributed to the article and approved the submitted version.

## Conflict of Interest

The authors declare that the research was conducted in the absence of any commercial or financial relationships that could be construed as a potential conflict of interest.

## Supplementary Material

Supplementary Figure 1 Correlation of TME scores with clinical characteristics. **(A)** No correlation between tumor immune score and lymph node metastasis in patients was found. There was no significant correlation between stromal score and T stage **(B)**, lymph node metastasis **(C)**, or gender **(D)**, respectively.

Supplementary Figure 2 Dot plots of GO and KEGG enrichment analysis for the 275 DEGs. **(A)** GO enrichment analysis for 275 TME-related DEGs, statistical threshold set at *P*-value < 0.05 with an FDR adjusted *P*-value < 0.05. (**B**) KEGG enrichment analysis for the 275 DEGs, statistical threshold set at p-value < 0.05 with an FDR adjusted *P*-value < 0.05.

Supplementary Figure 3 Results of survival analyses in male and female sub-cohorts of TCGA-HNSC. **(A, B)** No significant correlation between immune score and survival was observed in males or females (P>0.05). **(C–E)** The expression of all hub genes was significantly correlated with survival in the male group (P<0.005). **(F–H)** In the females, all hub genes’ expression was not associated with survival (P>0.05).

Supplementary Figure 4 Results of survival analyses of hub genes and gender in TCGA-HNSC patients with an OS >5 years. **(A, B)** Neither CCR8 nor P2RY14 gene expression levels in patients with survival beyond 5 years were significantly associated with prognosis (P>0.05). **(C)** The patients did not exhibit survival-related gender differences (P>0.05).

Supplementary Figure 5 Correlation of *CCR4* expression with immune cell fraction in tumor tissues. **(A)** There were significant differences in the fraction of some immune cells in the tumor tissue *(P*<0.05). **(B)** Correlation analysis of *CCR4* and immune cell content was used to validate the above differential analysis results.

Supplementary Figure 6 Correlation of *P2RY14* expression with immune cell fraction in tumor tissues. **(A)** There were significant differences in the fraction of some immune cells in the tumor tissue (*P*<0.05). **(B)** Correlation analysis of *P2RY14* and immune cell content was used to validate the above differential analysis results.
