# Supplementary figures and images for: CCR4, CCR8, and P2RY14 as Prognostic Factors in Head and Neck Squamous Cell Carcinoma Are Involved in the Remodeling of the Tumor Microenvironment
Source: Front Oncol. 2021 Feb 22;11:618187. doi: 10.3389/fonc.2021.618187 (PMC7937936; doi:10.3389/fonc.2021.618187)

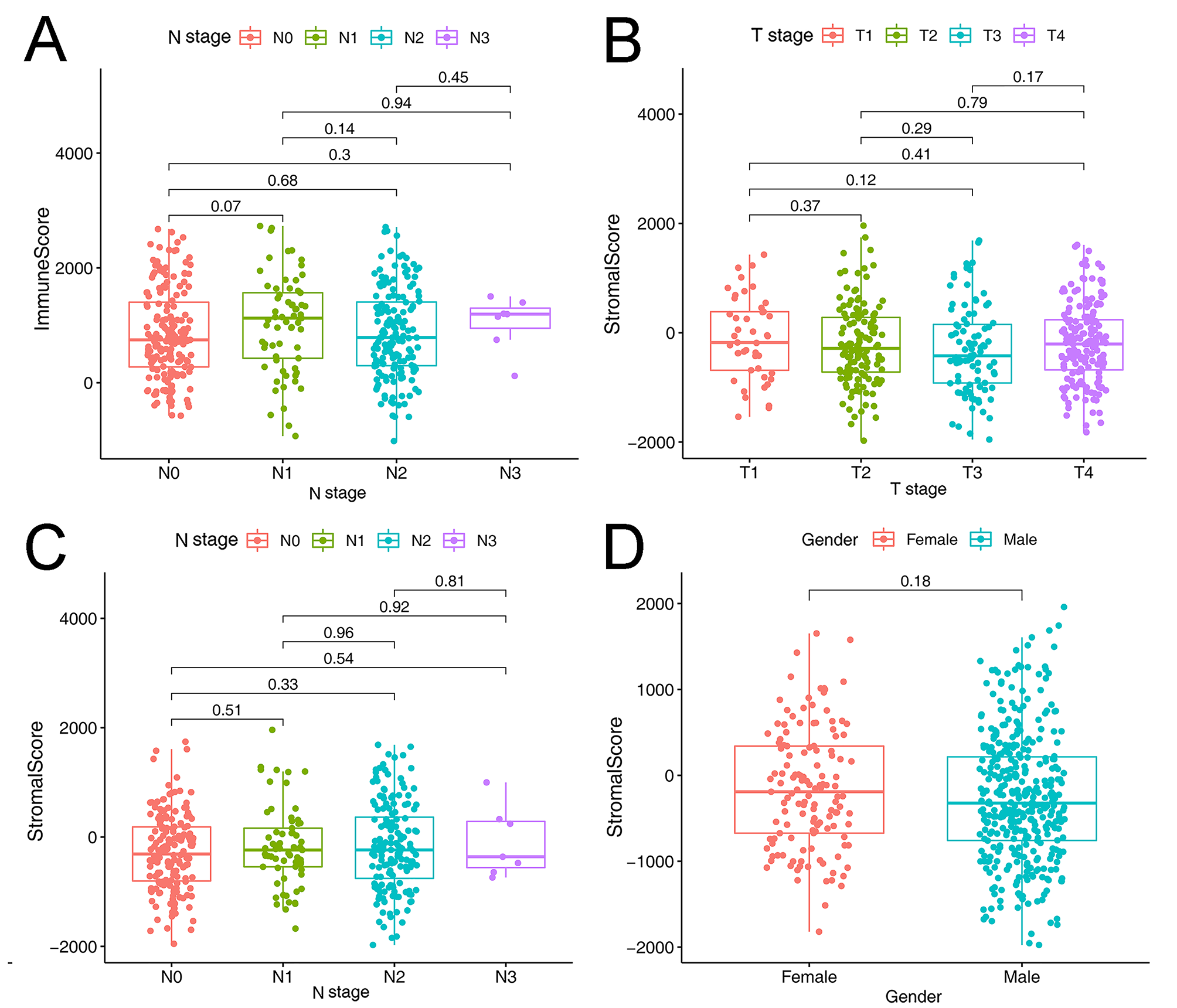

Supplement: Supplementary Figure 1 — Correlation of TME scores with clinical characteristics. (A) No correlation between tumor immune score and lymph node metastasis in patients was found. There was no significant correlation between stromal score and T stage (B), lymph node metastasis (C), or gender (D), respectively. [file Image_1.tif]

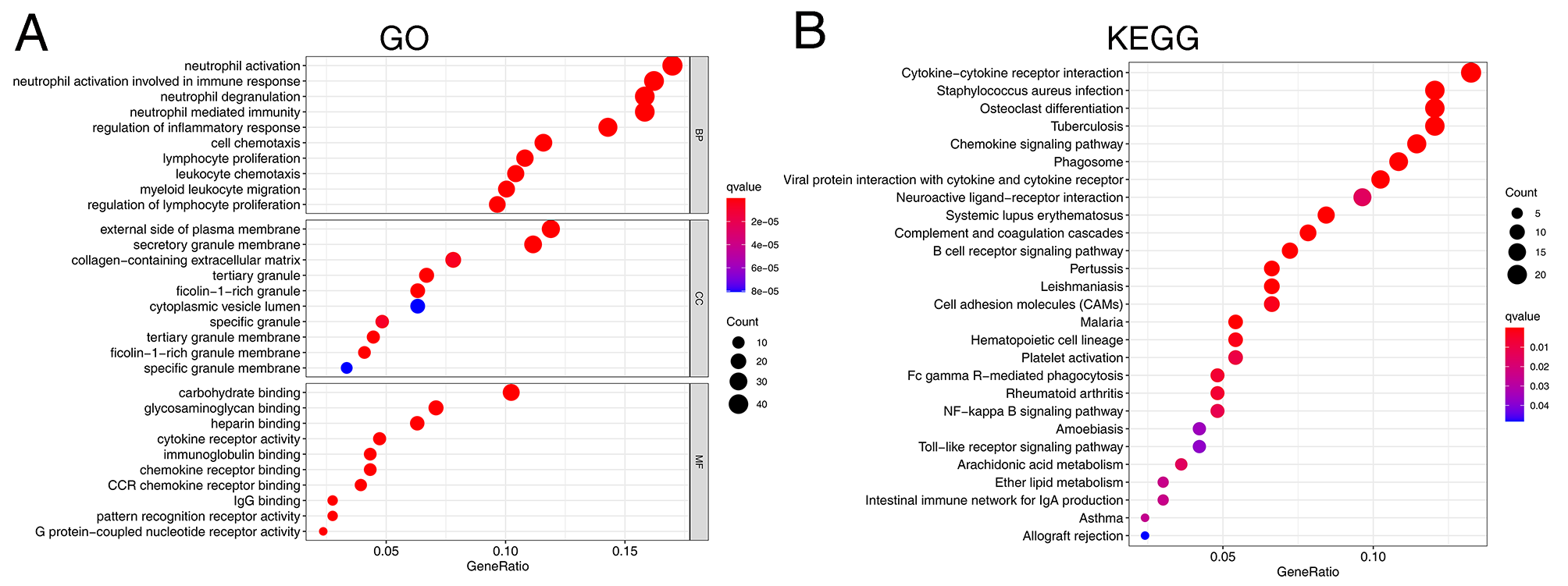

Supplement: Supplementary Figure 2 — Dot plots of GO and KEGG enrichment analysis for the 275 DEGs. (A) GO enrichment analysis for 275 TME-related DEGs, statistical threshold set at P-value < 0.05 with an FDR adjusted P-value < 0.05. (B) KEGG enrichment analysis for the 275 DEGs, statistical threshold set at p-value < 0.05 with an FDR adjusted P-value < 0.05. [file Image_2.tif]

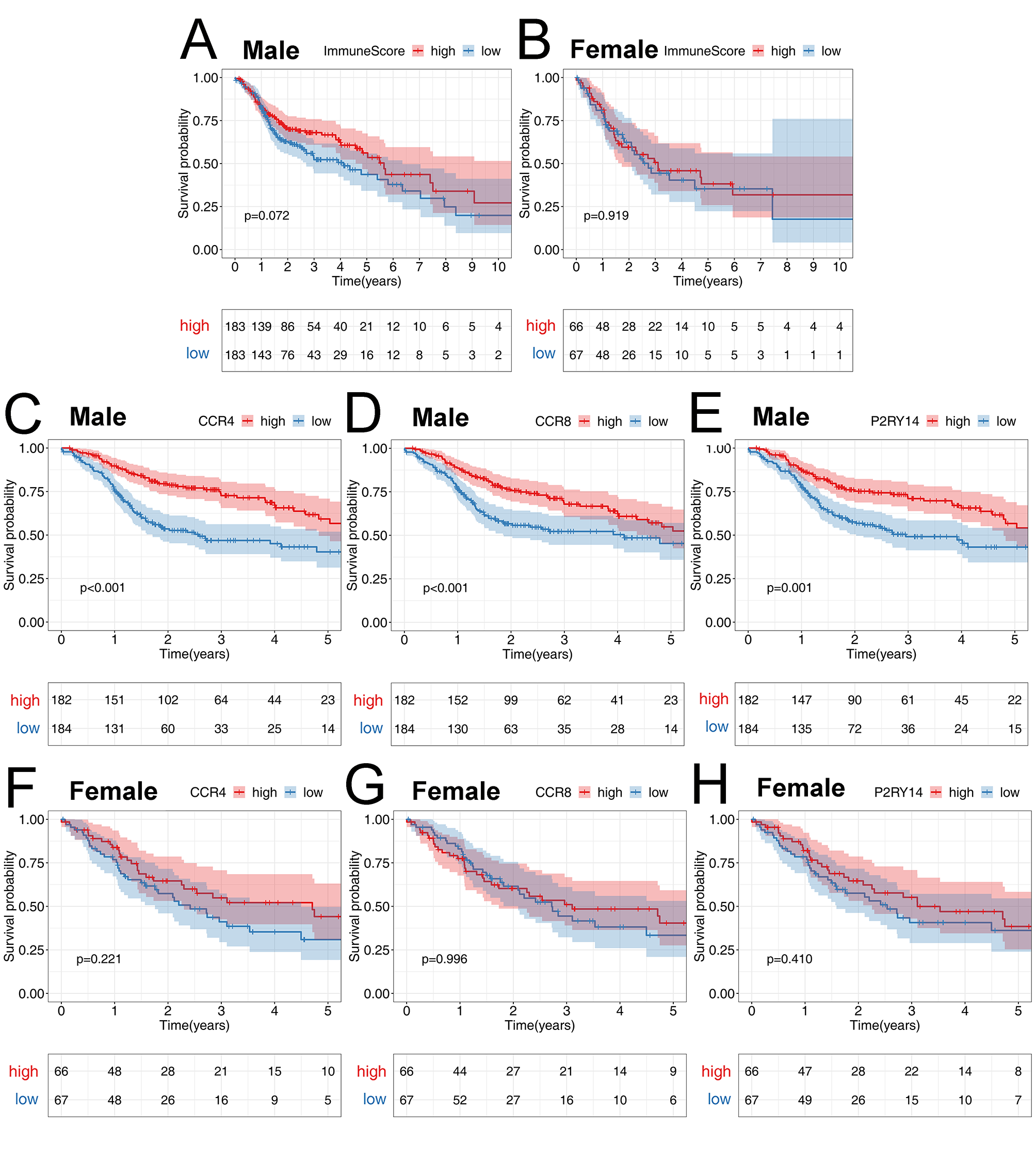

Supplement: Supplementary Figure 3 — Results of survival analyses in male and female sub-cohorts of TCGA-HNSC. (A, B) No significant correlation between immune score and survival was observed in males or females (P>0.05). (C–E) The expression of all hub genes was significantly correlated with survival in the male group (P<0.005). (F–H) In the females, all hub genes’ expression was not associated with survival (P>0.05). [file Image_3.tif]

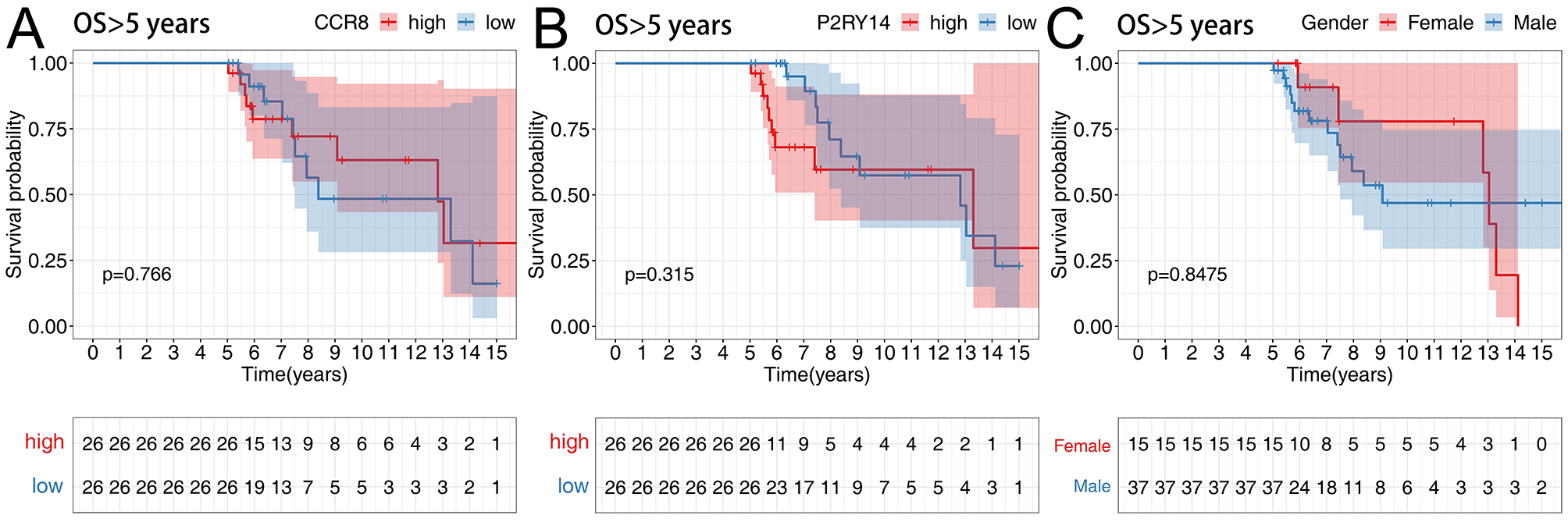

Supplement: Supplementary Figure 4 — Results of survival analyses of hub genes and gender in TCGA-HNSC patients with an OS >5 years. (A, B) Neither CCR8 nor P2RY14 gene expression levels in patients with survival beyond 5 years were significantly associated with prognosis (P>0.05). (C) The patients did not exhibit survival-related gender differences (P>0.05). [file Image_4.tif]

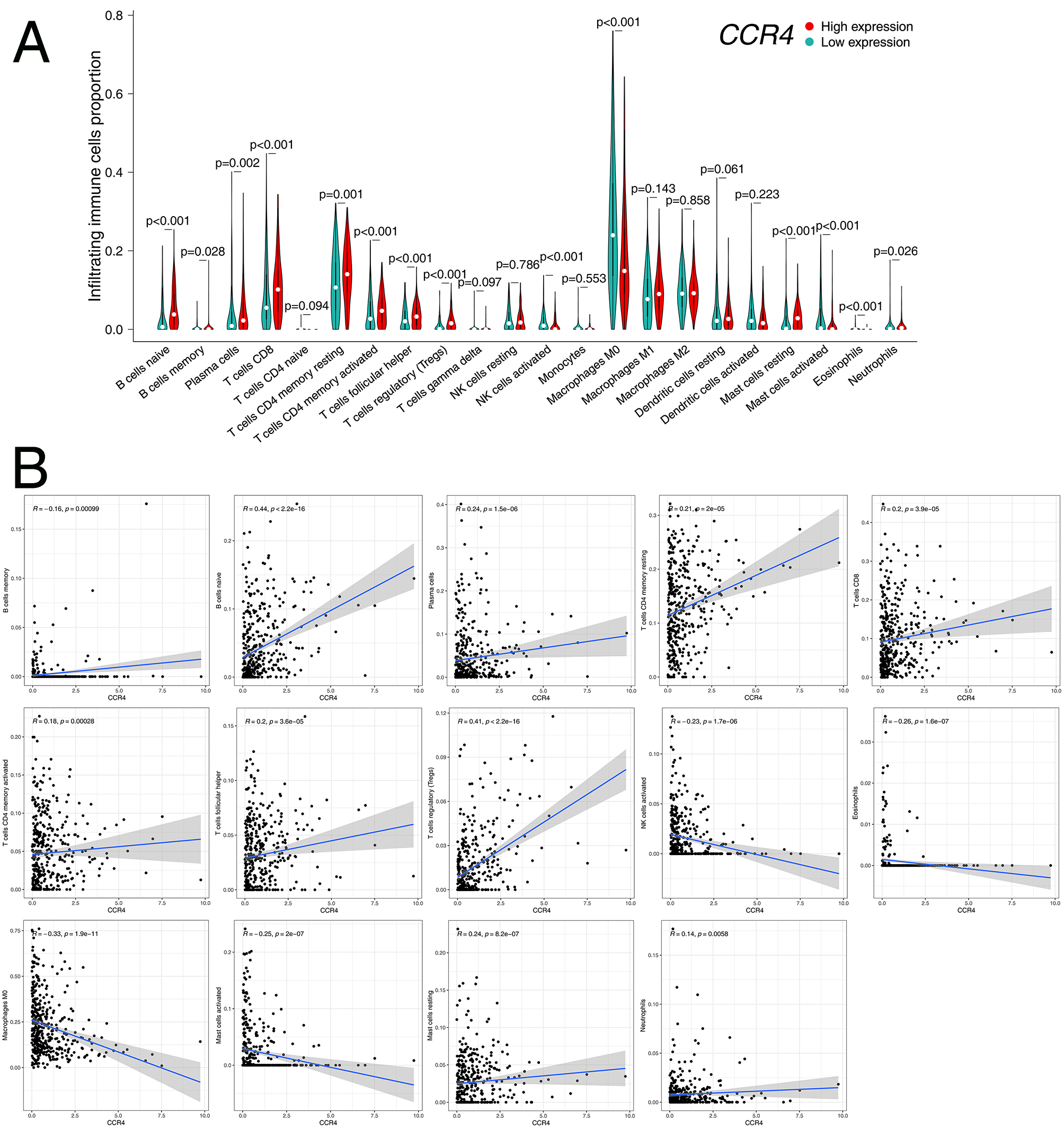

Supplement: Supplementary Figure 5 — Correlation of CCR4 expression with immune cell fraction in tumor tissues. (A) There were significant differences in the fraction of some immune cells in the tumor tissue (P<0.05). (B) Correlation analysis of CCR4 and immune cell content was used to validate the above differential analysis results. [file Image_5.tif]

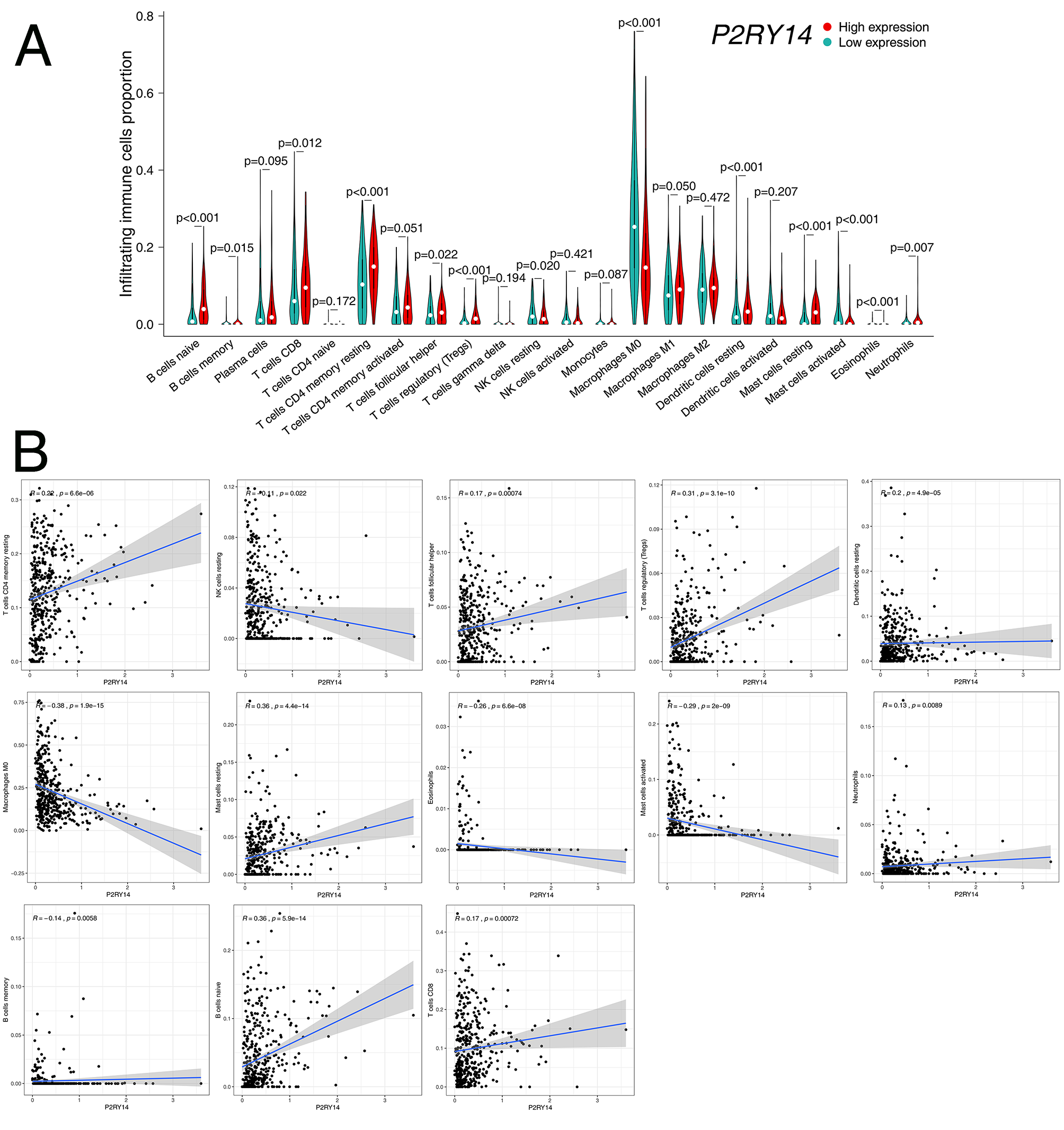

Supplement: Supplementary Figure 6 — Correlation of P2RY14 expression with immune cell fraction in tumor tissues. (A) There were significant differences in the fraction of some immune cells in the tumor tissue (P<0.05). (B) Correlation analysis of P2RY14 and immune cell content was used to validate the above differential analysis results. [file Image_6.tif]
