# Supplementary material for: CCR4, CCR8, and P2RY14 as Prognostic Factors in Head and Neck Squamous Cell Carcinoma Are Involved in the Remodeling of the Tumor Microenvironment
Source: Front Oncol. 2021 Feb 22;11:618187. doi: 10.3389/fonc.2021.618187 (PMC7937936; doi:10.3389/fonc.2021.618187)
Supplement: Supplementary file 7 [file Table_1.docx]

Table S1 Patient clinical and pathologic characteristics for TCGA-HNSC dataset.

|  | | TCGA-HNSC (n=499) |
| --- | --- | --- |
| Age | <65 | 310(62.12%) |
|  | >=65 | 189(37.88%) |
| Gender | Male | 366(73.35%) |
|  | Female | 133(26.65%) |
| Pathologic Stage | I-II | 94(18.84%) |
|  | III-IV | 337(67.54%) |
|  | Unknown | 68(13.63%) |
| T stage | I-II | 177(35.47%) |
|  | III-IV | 267(53.51%) |
|  | Unknown | 55(11.02%) |
| N stage | NO | 170(34.07%) |
|  | N1-3 | 236(47.29%) |
|  | Unknown | 93(18.64%) |
| M stage | M0 | 185(37.07%) |
|  | M1 | 1 (0.2%) |
|  | Unknown | 313(62.73%) |
| Grade | G1-2 | 359(71.94%) |
|  | G3-4 | 121(24.25%) |
|  | unknown | 19(3.81%) |
